# Ferritin RNA interference inhibits the formation of iron granules in the trophocytes of worker honey bees (*Apis mellifera*)

**DOI:** 10.1038/s41598-019-45107-0

**Published:** 2019-08-15

**Authors:** Chin-Yuan Hsu, Hsiao-Fan Lo, Navdeep S. Mutti, Gro V. Amdam

**Affiliations:** 1 Department of Biomedical Sciences, College of Medicine, Chang Gung University, Tao-Yuan, Taiwan; 2 Graduate Institute of Biomedical Sciences, College of Medicine, Chang Gung University, Tao-Yuan, Taiwan; 3 Department of Obstetrics and Gynecology, Chang Gung Memorial Hospital, Linkou, Taiwan; 4 School of Life Sciences, Arizona State University, Arizona, USA; 5 Corteva Agriscience, Indiana, USA; 6 Faculty of Environmental Sciences and Natural Resource Management, Norwegian University of Life Sciences, Aas, Norway

**Keywords:** Cell biology, Animal behaviour

## Abstract

Iron granules containing superparamagnetic magnetite act as magnetoreceptor for magnetoreception in honey bees. Biomineralization of iron granules occurs in the iron deposition vesicles of trophocytes and requires the participation of actin, myosin, ferritin2, and ATP synthase. The mechanism of magnetoreception in honey bees can be explored by suppressing the formation of iron granules. Toward this goal, we injected double-stranded RNA of *ferritin2* and *ferritin1* into newly emerged worker honey bees to knock down these genes via RNA interference. We confirmed that mRNA and protein production of the ferritins was inhibited, leading to immature iron granules. Downregulating *ferritin2* and *ferritin1*, moreover, leads to different deposition morphology of 7.5-nm diameter iron particles, indicating that the two genes play different roles in the formation of iron granules in worker honey bees.

## Introduction

Magnetoreception is a sense allowing an animal to detect the earth’s magnetic field to establish regional magnetic maps for navigation and positioning. Numerous studies demonstrate that honey bees have magnetoreception behavior. Applying an extra magnetic field changes the bees’ comb building and orientation and homing behaviors^1–3^. Gluing magnets to the anterior dorsal abdomen interface interferes with magnetic discrimination in choice experiments^4^. Free-flying honey bees can detect small static intensity fluctuations at the level of 26 nT against the earth-strength magnetic field^5^ and alternating fields of 430 μT at a frequency of 10 and 60 Hz^6^. In addition, the bees can be trained in discrimination assays to respond to local anomalies in a magnetic field^7,8^.

The magnetoreception of honey bees is further demonstrated by the finding of superparamagnetic magnetite in iron granules (IGs)^9,10^. Applying a magnetic field to the bee causes the IGs to shrink in the direction paralleled to the magnetic field and to elongate in the vertical direction to the magnetic field^10^. The changes in the IG’s confirmation by magnetic fields is posited to generate Ca^2+^ signals and signal transduction via fluctuation of cytoskeletons on iron deposition vesicles (IDVs)^10^. These events can allow honey bees to establish a magnetic map during orientation flights to facilitate future foraging behavior^10^. IGs, therefore, are proposed to act as the magnetoreceptor in honey bees and crucial to the bees’ orientation and homing^10^.

IGs are present in the IDVs of trophocytes which are located in the abdomen of honey bees^11^. IGs are derived from the aggregation of 7.5-nm diameter iron particles in the center of IDVs. 7.5-nm diameter iron particles are formed in the cloudy area beneath the inner membrane of IDVs^11^. IDVs are double-membrane vesicles with 10 nm wide space between the outer and inner membrane^11^. IGs are deposited from about 5 days to 25 days after adult emergence. When IGs are mature, they are filled to the inner membrane, leaving a 10 nm space between IGs and the outer membrane of IDVs^11^. An actin-myosin-ferritin transporter system participates in the formation of IGs^12^. Actin, myosin, ferritin 2, and ATP synthase are involved in this system^12^.

RNA interference (RNAi)-mediated gene knockdown has been used to study gene functions in different honey bee developmental stages, including embryos^13,14^, larvae^15,16^, pupae^17,18^, and fully developed adult honey bees^19–22^. Genes such as *E30 homeobox*, *CSP5*, *vitellogenin*, *insulin receptor substrate*, *am-tra2*, *am18w*, *ultraspiracle*, *octopamine receptor*, and *DNA methyl-transferase* have been successfully knocked down in honey bees by double-stranded RNA (dsRNA) injection^13,14,17,19,22–26^.

Ferritin is a hollow globular protein that stores iron and releases it. Each ferritin can store about 4500 iron (Fe^+^3) ions^27^. Ferritins are composed of heavy chains and light chains. Heavy chains are important for Fe^+^2 oxidation and light chains assist in core formation^27^. In honey bees (*Apis mellifera*), the heavy chain is called ferritin1 or Fer1HCH and the light chain is called ferritin2 or Fer2LCH. The amino acids sequences of ferritin2 (XP_624076.1) and ferritin1 (XP_016771737.1) is 23% degree of identity. In this study, we knocked down the genes for *ferritin2* and *ferritin1* to test whether ferritins RNAi can be used to inhibit or change IGs formation. The technique developed here may be used to further explore magnetoreception in honey bees.

## Methods

### The preparation of dsRNA toward *ferritin2*, *ferritin1*, and *green fluorescent protein* (*GFP*, control)

The primers were designed based on the nucleotide sequences available in GenBank: *ferritin2* (Fer2LCH) (XM_624073.4): forward 5′-ATTTTTGGCAACTGCCTCTG-3′, reverse 5′-ATTCTCGAACACGGTCTGCT-3′; *ferritin1* (Fer1HCH) (XM_016916248.1): forward 5′-CCCCGTCGATTAAAGTACGA-3′, reverse 5′-GCATGTTCTCTTTCTTCTGTAGCA-3′; *GFP*: forward 5′-GAGATACCCAGATCAT-3′, reverse 5′-GATGATATTCACCACTT-3′. Primers were fused with the T7 promoter sequence. Total RNA was isolated from the trophocytes and oenocytes of three worker bees at 3 days after emergence using TRIzol (15596018; Invitrogen, Carlsbad, CA, USA) following the manufacturer’s instructions. The complementary DNA (cDNA) was synthesized by using Superscript III First-Strand Synthesis System for RT-PCR (18080-051; Invitrogen, Carlsbad, CA, USA). The cDNA was transferred into *E*. *coli* by using Topo TA cloning Kit for sequencing (450030, Invitrogen, Carlsbad, CA, USA). The plasmid was isolated with QIAprep Spin Miniprep Kit (27104, Qiagen, Valencia, CA, USA). The double-stranded (dsDNA) was produced by PCR using the T7 primers and purified by QIA Quick Gel Extraction Kit (28704, Qiagen, Valencia, CA, USA). Finally, dsRNA was synthesized and purified by using AmpliScribe^TM^ T7-Flash^TM^ Transcription Kit (ASF3257, Epicentre Biotechnologies, Madison, WI, USA), and diluted with nuclease-free water to a final concentration of 5 μg/μl.

### The microinjection of *ferritin2*, *ferritin1*, and *GFP* dsRNA

The microinjection of *ferritin2*, *ferritin1*, and *GFP* dsRNA into hemolymph was followed as Amdam’s method with minor modification^19^. Newly emerged worker honey bees were immobilized on a disc of bee wax with two crossed metal needles at room temperature. The bees were injected with 1 μl nuclease free water or 1 μl dsRNA solution (5 μg/μl) with a microinjector (Femtojet, Eppendorf). Microinjection was performed on the dorsum of the abdomen between the 1st and 2nd abdominal segment with glass needles. Care was taken to minimize mechanical damage to the bees. Individuals showing hemolymph leakage after microinjection were discarded. Successfully injected bees were housed in a cage (15 × 10 × 12 cm^3^) for 1 h before moving into an incubator set to 34 °C (NK system, Nippon, Japan). The worker bees were fed honey and fresh pollen grains mixed with honey (3:1) every day^28^.

### Quantitative real-time polymerase chain reaction (qPCR) analysis

Trophocytes and oenocytes were isolated from two worker bees 6 days after emergence. Total RNA was extracted from these cells using Trizol^®^ Reagent (15596018; Invitrogen, CA, USA). RNA concentration and quality were determined using a Synergy^TM^ HT multi-mode microplate reader (7091000; BioTek). The cDNA synthesis was performed using an iScript™ cDNA synthesis kit (170-8891; Bio-Rad Laboratories, CA, USA). Each reaction contained 1 μg of total RNA in a 20 µl reaction volume. The qPCR was performed using a CFX connect RT-PCR detection system (Bio-Rad Laboratories) and each reaction contained 0.5 µl of 10 µM of each primer, 12.5 µl of SYBR Green (170-8882; Bio-Rad Laboratories), 1 µl of cDNA, and 10.5 µl of ddH_2_O in a final volume of 25 μl. Primer sequences were noted above. The *actin* gene was used as reference gene. Ten replicates were performed, and 20 worker bees in total were used in each group.

### Western blotting

Trophocytes and oenocytes were isolated from two worker bees, homogenized in 100 μl of radioimmunoprecipitation (RIPA) buffer containing protease inhibitors (11697498001; Roche Applied Science, Indianapolis, IN, USA), and centrifuged at 5,000 *g* for 10 min at 4 °C. The protein concentration of resulting supernatant was determined using a protein assay reagent (500-0006; Bio-Rad Laboratories, Hercules, CA, USA). Proteins (30 μg) from the supernatant were resolved by sodium dodecyl sulfate-polyacrylamide gel electrophoresis (SDS/PAGE) on 10–15% polyacrylamide gels and transferred to polyvinylidene fluoride (PVDF) membranes. After blocking for 1 h at 25 °C, membranes were first incubated with primary antibodies against ferritin2 (1:1,000; produced in-house) or tubulin (ab6046, 1:10,000; Abcam, Cambridge, MA, USA) and then probed with the appropriate horseradish peroxidase-conjugated secondary antibody (1:10,000). Immunoreactive proteins were detected using a chemiluminescence method (PerkinElmer, Covina, CA, USA) and analyzed using Image J software (NIH, Bethesda, MA, USA). The protein production levels were normalized to tubulin^28^. Ten replicates were performed, and thus 20 worker bees in total were used in each group.

### Transmission electron microscopy (TEM)

Worker bees without RNAi (control group 1), with *GFP* RNAi (control group 2), and with *ferritins* RNAi (experimental group) at 6, 8, 10, 12, 14, and 20 days after emergence were fixed in 2.5% glutaraldehyde in a 0.1 M phosphate buffer containing 0.35 M sucrose (pH 7.4) for 30 min at 25 °C, and postfixed in 1% osmium tetroxide in a 0.1 M phosphate buffer with 0.35 M sucrose (pH 7.4) for 2 h. Trophocytes were dehydrated through an ethanol series and embedded in Spurr’s resin. Thin sections (60–90 nm in thickness) were cut with a diamond knife, stained with uranyl acetate and lead citrate, and then examined using a TEM system (JEOL JEM-2000EXII; Tokyo, Japan) operating at an accelerating voltage of 100 kV^12^.

### IGs formation assay

IGs formation was evaluated according to the IG area/IDV area ratio from TEM images. The IG area and IDV area were analyzed using Photoshop (CS6)^29^. Five replicates were performed, and thus 5 worker bees in total were used in each group. Ten IDVs in each bee were used to evaluate IGs formation. Thus, the results for IGs formation were derived from 50 IDVs in each group.

### Statistical analysis

Differences in the mean values among the three treatment groups were determined by one-way ANOVA and by Tukey’s HSD for pairwise comparisons. SPSS software was used for statistical analysis. A *p*-value of less than 0.05 was considered statistically significant.

## Results

### *Ferritin2* RNAi inhibits the mRNA and protein production of ferritin2

**Figure 1:**
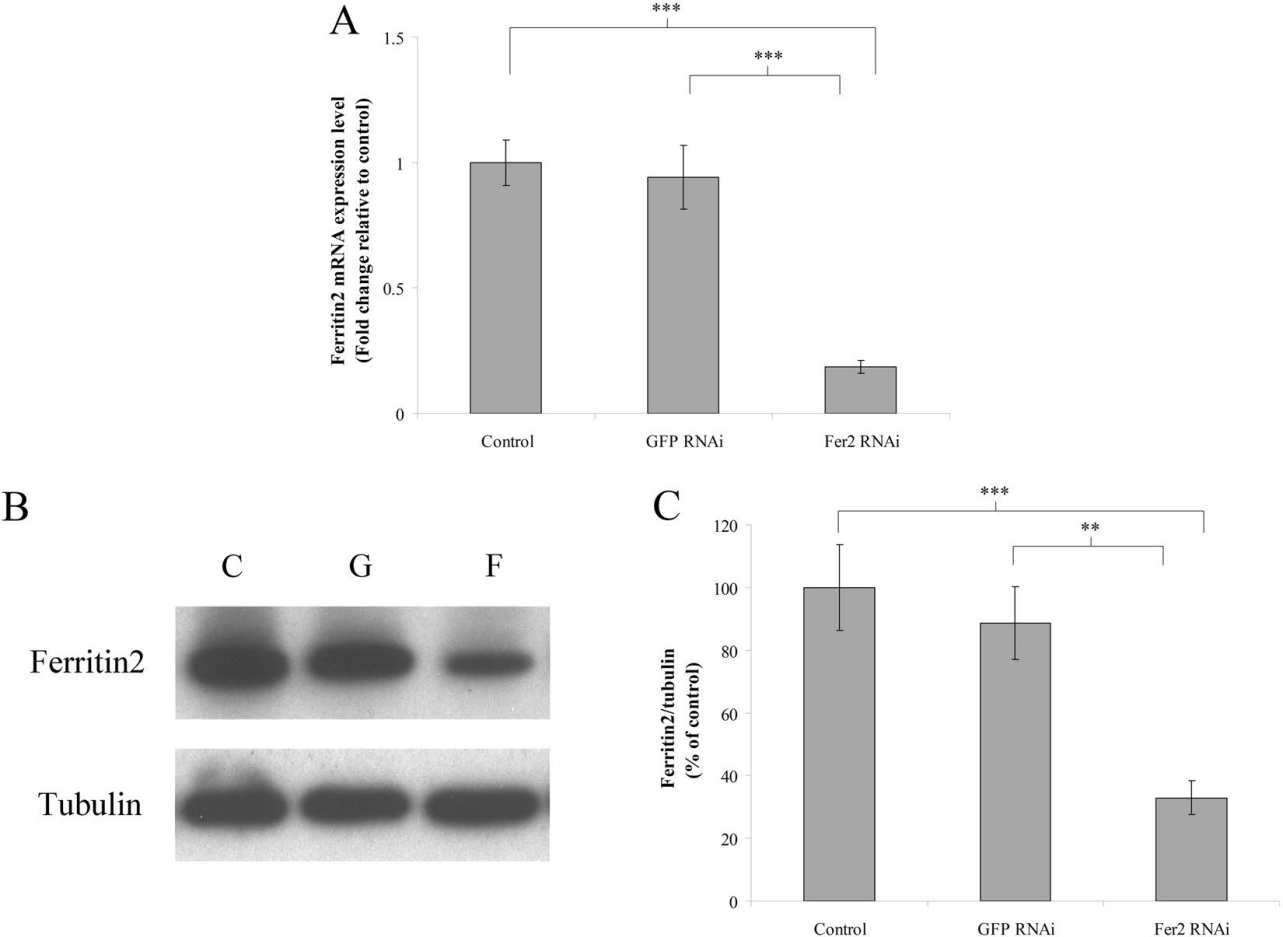
The production of ferritin2 mRNA and protein in worker bees at 6 days after *ferritin2* RNAi. (**A**) The mRNA production of *ferritin2* in trophocytes and oenocytes was measured via qPCR. Actin served as the loading control. The results were normalized to the water control and are shown as fold changes, representing the mean ± standard error of the means (SEMs) (*n* = 10). Fer2, ferritin2. (**B**) Ferritin2 protein was analyzed by western blotting. Tubulin served as the loading control. C, control; G, *GFP* dsRNA; F, *ferritin2* RNAi. (**C**) The production levels of ferritin2 were normalized to the control. The results were expressed as percentages and presented as the means ± SEMs (*n* = 10). Fer2, ferritin2. Asterisk indicates a statistically significant difference (**P < 0.01; ***P < 0.001; one-way ANOVA).

To evaluate the effect of *ferritin2* RNAi, we assayed the *ferritin2* mRNA and protein levels in the trophocytes and oenocytes of worker bees at 6 days after *ferritin2* or *GFP* dsRNA injection. We found that worker bees subjected *ferritin2* RNAi had lower *ferritin2* mRNA levels than the water control and the *GFP* RNAi control (*n* = 10, *P* < 0.05; Fig. 1A), indicating that *ferritin2* RNAi suppressed *ferritin2* mRNA production. Worker bees with *ferritin2* gene knockdown also had lower ferritin2 protein levels than the water control and the *GFP* RNAi control (Fig. 1B). Statistical analyses revealed that ferritin2 protein levels were significantly different from the water control and the *GFP* RNAi control (*n* = 10, *P* < 0.05; Fig. 1C) indicating that *ferritin2* gene knockdown also suppressed ferritin2 protein production.

### *Ferritin2* RNAi inhibits the formation of IGs

**Figure 2:**
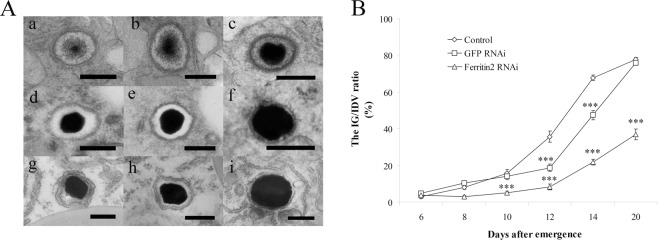
The morphology of IGs in the trophocytes of worker bees after *ferritin2* RNAi. (**A**) The morphology of IGs on 12 (a), 14 (b), and 20 (c) days after *ferritin2* dsRNA injection, 12 (d), 14 (e), and 20 (f) days after *GFP* dsRNA injection, and 12 (g), 14 (h), and 20 (i) days after nuclease free water injection. Scale bar in images a–i, 0.3 μm. (**B**) Size changes of IGs in different days after *ferritin2* dsRNA injection. Bar represents the means ± SEMs (*n* = 50). ◊, control; □, *GFP* dsRNA; Δ, *ferritin2* RNAi. Asterisk indicates a statistically significant difference (*** < 0.001; one-way ANOVA).

To evaluate the effect of *ferritin2* knockdown on IGs formation, we examined the morphology of IGs at the 6, 8, 10, 12, 14, and 20 days after dsRNA injection. We found that the IGs of worker bees with *ferritin2* knockdown had decreased iron mineralization at 12, 14, and 20 days after *ferritin2* RNAi (Fig. 2A,a–c), as compared to the *GFP* RNAi control (Fig. 2A,d–f) and the water control (Fig. 2A,g–i). Statistical analyses revealed that iron mineralization in the worker bees with *ferritin2* knockdown was significantly different from the *GFP* RNAi control and the water control (*n* = 50, *P* < 0.05; Fig. 2B) indicating that *ferritin2* RNAi inhibited the formation of IGs. The *GFP* RNAi control is also significantly different from the water control at 12 and 14 days.

### *Ferritin1* RNAi inhibits the mRNA production of ferritin1

**Figure 3:**
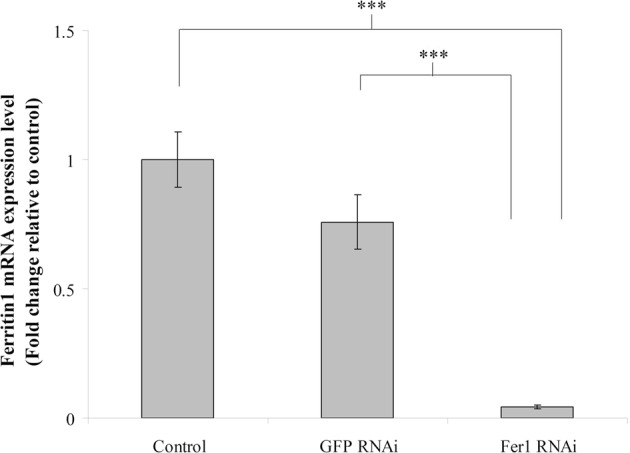
The production of ferritin1 mRNA in worker bees at 6 days after *ferritin1* RNAi. The mRNA production of *ferritin1* in trophocytes and oenocytes was measured via qPCR. Actin served as the loading control. The results were normalized to those of the water control (control) and are shown as fold changes, representing the mean ± SEMs (*n* = 10). Fer1, ferritin1. Asterisk indicates a statistically significant difference (***P < 0.001; one-way ANOVA).

To evaluate the effect of *ferritin1* RNAi, we assayed the *ferritin1* mRNA levels in the trophocytes and oenocytes of worker bees at 6 days after *ferritin1* or *GFP* dsRNA injection. We found that worker bees treated *ferritin1* RNAi had lower *ferritin1* mRNA levels than the water control and the *GFP* RNAi control. Statistical analyses revealed that the mRNA levels of *ferritin1* were significantly different the water control and the *GFP* RNAi control (*n* = 10, *P* < 0.05; Fig. 3) indicating that *ferritin1* RNAi suppressed *ferritin1* mRNA production.

### *Ferritin1* RNAi inhibits the formation of IGs

**Figure 4:**
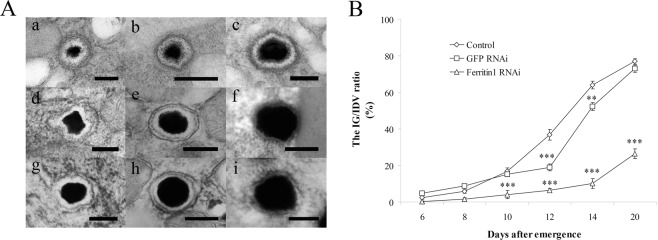
The morphology of IGs in trophocytes after *ferritin1* RNAi. (**A**) The morphology of IGs on 12 (a), 14 (b), and 20 (c) days after *ferritin1* dsRNA injection, 12 (d), 14 (e), and 20 (f) days after *GFP* dsRNA injection, and 12 (g), 14 (h), and 20 (i) days after nuclease free water injection. Scale bar in images a–i, 0.2 μm. (**B**) Size changes of IGs in different days after *ferritin1* dsRNA injection. Bar represents the means ± SEMs (*n* = 50). ◊, control; □, *GFP* dsRNA; Δ, *ferritin1* RNAi. Asterisk indicates a statistically significant difference (** < 0.01; *** < 0.001; one-way ANOVA).

To evaluate the effect of *ferritin1* knockdown on IGs formation, we examined the morphology of IGs at the 6, 8, 10, 12, 14, and 20 days after dsRNA injection. We found that the IGs of worker bees with *ferritin1* knockdown had decreased iron mineralization at 12, 14, and 20 days after *ferritin1* RNAi (Fig. 4A,a–c), as compared to the *GFP* RNAi control (Fig. 4A,d–f) and the water control (Fig. 4A,g–i). Statistical analyses revealed that iron mineralization in the worker bees with *ferritin1* knockdown was significantly different from the *GFP* RNAi control and the water control (*n* = 30, *P* < 0.05; Fig. 4B) indicating that *ferritin1* RNAi inhibited the formation of IGs. The *GFP* RNAi control is also significantly different from the water control at 12 and 14 days.

### The different deposition morphology of 7.5-nm diameter iron particles from *ferritin2* and *ferritin1*RNAi

**Figure 5:**
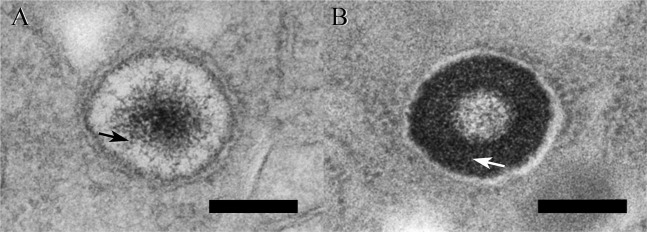
The deposition morphology of 7.5-nm diameter iron particles in the trophocytes of worker bees at 6 days after by *ferritins* dsRNA injection. (**A**) An IDV at 6 days after *ferritin2* RNAi. Scale bar, 0.2 μm. Arrow, 7.5 nm particles. (**B**) An IDV at 6 days after *ferritin1* RNAi. Scale bar, 0.2 μm. Arrow, 7.5 nm particles.

The deposition morphology of 7.5-nm diameter iron particles via *ferritin2* RNAi was different from that of *ferritin1* RNAi at 6 days after dsRNA injection, indicating that *ferritin2* and *ferritin1* may play different roles in the formation of IGs (Fig. 5).

## Discussion

Actin, myosin, ferritin2, and ATP synthase are involved in the actin-myosin-ferritin transporter system which participates in the formation of IGs in the IDVs of trophocytes^12^. In this study, we demonstrate that the mRNA and/or protein production of ferritin2 or ferritin1 and the formation of IGs in trophocytes can be inhibited by *ferritin2* or *ferritin1* RNAi. This technique may be used to further explore the magnetoreception of honey bees.

### *Ferritins* RNAi inhibits the mRNA and/or protein production of ferritins

Ferritin packaged with iron ions is a 7.5-nm spherical iron particle in IDVs. *Ferritin2* RNAi inhibits the mRNA and protein production of ferritin2. Likewise, *ferritin1* RNAi inhibits the mRNA production of ferritin1. These phenomena are consistent with previous studies showing that the mRNA production of honey bees can be inhibited by RNAi^19–22^.

### *Ferritin2* and *ferritin1* RNAi inhibit the formation of IGs

7.5-nm diameter iron particles are formed in the cloudy area beneath the inner membrane of IDVs and move to the center of IDVs for aggregation which forms IGs^11^. The IGs formation is proposed and summarized as follows: Fe^2+^ from the cytoplasm is transported into the acidic space (pH < 7) between the outer and inner IDV membranes via a transporter protein. An H^+^/Fe^2+^ antiporter on the inner IDV membrane then simultaneously transports one molecule of Fe^2+^ into the acidic space and two molecules of H^+^ out of the acidic space to maintain a pH < 7 in the acidic space and a pH > 7 in the alkaline lumen of IDVs. Fe^2+^ then becomes partially oxidized to Fe^3+^, and Fe^2+^/Fe^3+^ is integrated into apoferritin in the cloudy layer of IDVs to form 7.5-nm spherical iron particles (ferritin)^11,12,30^. Then, ferritin attached to myosin is transported along an actin chain to the center of IDVs in a manner that is dependent on Ca^2+^ and ATP^12^. This transporter system is called the actin-myosin-ferritin transporter system. Actin, myosin, and ferritin2 are involved in actin-myosin-ferritin transporter system^12^. *Ferritin2* RNAi inhibited the formation of IGs demonstrated that *ferritin2* has a relationship with the formation of 7.5-nm diameter iron particles and *ferritin2* is involved in the formation of IGs. This finding is consistent with a previous study^12^. In addition, *ferritin1* RNAi inhibited the formation of IGs demonstrated that *ferritin1* has a relationship with the formation of 7.5-nm diameter iron particles and *ferritin1* is involved in the formation of IGs. The technique developed here can be used to further explore magnetoreception in honey bees. The *GFP* RNAi control is also significantly different from the water control at 12 and 14 days. The most likely reason is that *GFP* dsRNA interferes the synthesis of ferritins at 12 and 14 days, a period of mass synthesis of ferritins for iron deposition.

### *Ferritin2* and *ferritin1* play different roles in the formation of IGs

The image of IGs after *ferritin2* knockdown showed that the accumulation of 7.5-nm diameter iron particles in the center of IDVs of *ferritin2* RNAi bees is similar to that of the water control^11^. However, this accumulation in *ferritin2* RNAi bees is slower than that in the water control due to 7.5-nm diameter iron particles deposited slowly^11^. This phenomenon shows that *ferritin2* may be involved in the formation of 7.5-nm diameter iron particles. The deposition morphology of 7.5-nm diameter iron particles after *ferritin1* knockdown showed that most of 7.5-nm diameter iron particles accumulated on the periphery of the center of IDVs and are different from that of *ferritin2* RNAi. This phenomenon shows that *ferritin1* may have a relationship with the transportation of 7.5-nm diameter iron particles. Ferritin1 may be associated with the tail of myosin^12^.

These phenomena were only observed at the early stage of *ferritin* RNAi. The most likely reason is that the dsRNA dosage of one injection did not inhibit gene expression and the formation of IGs throughout the entire observation period.

## Supplementary information

Supplementary information
