# Supplementary material for: Ferritin RNA interference inhibits the formation of iron granules in the trophocytes of worker honey bees (Apis mellifera)
Source: Sci Rep. 2019 Aug 15;9:10098. doi: 10.1038/s41598-019-45107-0 (PMC6695493; doi:10.1038/s41598-019-45107-0)
Supplement: Supplementary file 1 — Supplementary information [file 41598_2019_45107_MOESM1_ESM.pdf]

Ferritin RNA interference inhibits the formation of iron granules in the trophocytes of worker honey bees (*Apis mellifera*)

Chin-Yuan Hsu, Hsiao-Fan Lo, Navdeep S. Mutti, Gro V. Amdam

Ferritin2

Tubulin

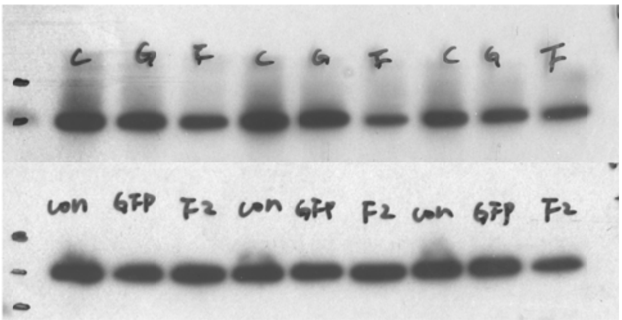

Figure S1 The original images of figure 1B. The figure 1B was cropped from the middle pattern of this figure. C and Con, control; G and GFP, *GFP* dsRNA; F and F2, *ferritin2* RNAi.
